# Brain-derived neurotrophic factor involved epigenetic repression of UGT2B7 in colorectal carcinoma: A mechanism to alter morphine glucuronidation in tumor

**DOI:** 10.18632/oncotarget.16251

**Published:** 2017-03-16

**Authors:** Zi-Zhao Yang, Li Li, Ming-Cheng Xu, Hai-Xing Ju, Miao Hao, Jing-Kai Gu, Zai-Jie Jim Wang, Hui-Di Jiang, Lu-Shan Yu, Su Zeng

**Affiliations:** ^1^ Laboratory of Pharmaceutical Analysis and Drug Metabolism, Zhejiang Province Key Laboratory of Anti-Cancer Drug Research, College of Pharmaceutical Sciences, Zhejiang University, Hangzhou, 310058, China; ^2^ Department of Pharmacy, Zhejiang Hospital, Zhejiang Provincial Key Lab of Geriatrics, Hangzhou 310013, China; ^3^ Department of Colorectal Surgery, Zhejiang Provincial Tumor Hospital, Hangzhou, 310022, China; ^4^ Science Research Center, China-Japan Union Hospital of Jilin University, Changchun 130033, China; ^5^ School of Life Sciences, Jilin University, Changchun, 130012, China; ^6^ Department of Biopharmaceutical Sciences and Cancer Center, University of Illinois at Chicago, Chicago, Illinois 60612, USA

**Keywords:** UGT2B7, BDNF, morphine tolerance, colorectal carcinoma, epigenetics

## Abstract

Uridine diphosphate-glucuronosyltransferase (UGT) 2B7, as one of significant drug enzymes, is responsible on the glucuronidation of abundant endobiotics or xenobiotics. We here report that it is markedly repressed in the tumor tissues of colorectal carcinoma (CRC) patients. Accordingly, morphine in CRC cells will stimulate the expression of its main metabolic enzyme, UGT2B7 during tolerance generation by activating the positive signals in histone 3, especially for trimethylated lysine 27 (H3K4Me3) and acetylated lysine 4 (H3K27Ac). Further study reveals that brain-derived neutrophilic factor (BDNF), a secretory neurotrophin, enriched in CRC can interact and inhibit UGT2B7 by primarily blocking the positive signals of H3K4Me3 as well as activating H3K27Ac on the promoter region of UGT2B7. Meanwhile, BDNF repression attributes to the sensitizations of main core factors in poly-comb repressive complex (PRC) 1 rather than PRC2 as the reason of the depression of SUZ12 in the later complex. Besides that, the productions of two main morphine glucuronides are both increased in the BDNF deficient or TSA and BIX-01294 treated morphine tolerance-like HCT-116 cells. On the same condition, active metabolite, morphine-6-glucuronide (M6G) was accumulated more than inactive M3G. Our findings imply that enzymatic activity enhancement and substrate regioselective catalysis alteration of UGT2B7 may release morphine tolerance under the cure of tumor-induced pain.

## INTRODUCTION

Colorectal carcinoma (CRC) may induce drastic pain inevitably. To release the tumor-induced pain, opioid potent drug, morphine would be primarily used though it may approach to severe side-effects such as dependence, opioid-dependent hyperalgesia (OIH) or tolerance [1–3]. In human body, morphine is metabolized mainly in the liver, but it can still be reabsorbed and metabolized in the colorectum [4]. It is worth to mention that one of its glucuronide metabolites, morphine-6-glucuronide (M6G), which is catalyzed by uridine diphosphate-glucuronosyltransferase 2B7 (UGT2B7), performs more analgesic effects than morphine-3-glucuronide (M3G) or morphine prototype [5]. After treated with the same doses of morphine and M6G, tolerant mice need expose more of the later one to recover the initial status [6]. Interestingly, M3G can prevent from M6G-medaited tolerance by blocking its antinociceptive effect [6, 7]. Those facts support both M3G and M6G would be significant in morphine tumor analgesia induced tolerance.

Recent research illustrates the expression of UGTs can be regulated under epigenetic changes or posttranslational modifications, such as DNA hyper methylation, aberrant histone modification and phosphorylation. Some of those regulations can selectively transform the catalyzing activities to their substrates [8–10]. For example, DNA hyper methylations mediated UGT1A1 regression would contribute to irinotecan active metabolite, 7-ethyl-10-hydroxycamptothecin (SN-38) inactivation and cytotoxicity increase in human CRC cells. Meanwhile, combining usage of the inhibitors of DNA methyltransferase (DNMT) or histone deacetylase (HDAC) including 2′-deoxy-5-azacytidine (DAC) and trichostatin A (TSA) can reverse this reaction [11]. Accordingly, our preliminary data show that UGT2B7 depressed in CRC. Thus, whether epigenetic variants of UGT2B7 can induce aberrant morphine glucuronidation became a hypothesis in our research.

As preserving the survival of several populations of central neurons [12, 13], brain-derived neurotrophic factor (BDNF) now has been discovered with much higher expression level in colorectal carcinoma tissues [14, 15]. Many clues elucidate it has a tight connection to morphine antinociception. Chronic morphine explosion can dominantly enrich the negative or silencing signal, trimethylated lysine 27 in histone 3 (H3K27Me3), on the promoter of BDNF at mice ventral tegmental area (VTA) region [16], then down-regulate nuclear receptor related-1 (NURR1) expression [17]. Moreover, knocking BDNF out in the microglia of gene-targeted mice is able to preserve Cl (−) homeostasis and not to develop hyperalgesia during morphine treatment [18]. Similarly, suppressive effect of it will enhance the ability of morphine to increase dopamine (DA) neuron excitability and promote euphoria of mice. All these prove BDNF should be a negative modulator of morphine action [19]. However, very little is known about the relationship between BDNF enrichment and UGT2B7 suppression in CRC. To address these questions and explore the mechanism behind the phenomena series of assays were designed and performed.

## RESULTS

### UGT2B7 specific depresses in CRC

**Figure 1:**
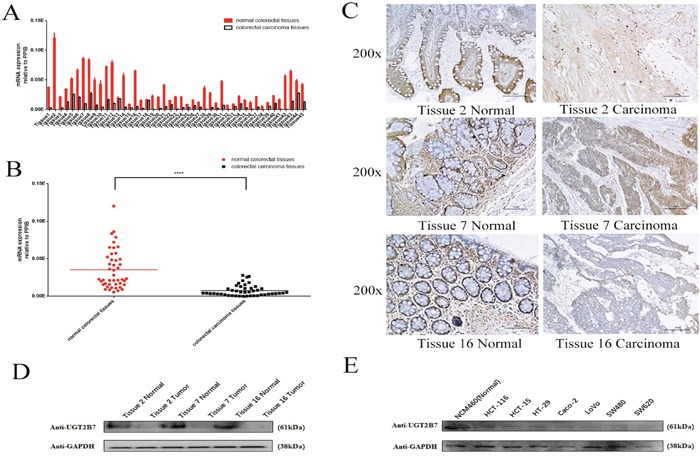
UGT2B7 is specifically repressed in CRC **(A)** mRNA expression of UGT2B7 in 45 pairs of RCC and adjacent non-tumor tissues normalized by the housekeeping gene of peptidylprolyl isomerase B (PPIB). **(B)** Microarray statistical analysis of UGT2B7 expression differences in 45 pairs of samples. **(C)** Representative images of immunohistochemistry staining for UGT2B7 protein on three selected pairs of RCC and adjacent non-tumor tissues with the largest differences, including No.2, No.7 and No.16. **(D)** UGT2B7 protein expression in these three pairs of samples. Housekeeping gene of glyceraldehyde-3-phosphate dehydrogenase (GAPDH) was used to normalize the data. **(E)** UGT2B7 protein expression in NCM460, human normal colorectal cells and different CRC cell lines. GAPDH was used as control to normalize the data. Results were presented from triplicated treatments as means ± SEM. ****P<0.0001. Unpaired student's t-test was used to calculate the P value.

We primary determined the UGT2B7 expression in 45 pairs of tumors and adjacent normal tissues in CRC patients (Supplementary Table 1). Consistent with the data analyzed by SYBR-Green quantitative polymerase chain reaction (qPCR), UGT2B7 mRNA expression was significantly declined in tumor tissues compared to adjacent normal tissues (Figure 1A, Figure 1B). After measuring the differences presented by each pair of tissues, three of them (Tissue No.2, No. 7 and No.16) were selected for further investigation. Immunohistochemistry and blotting images reflected a same trend as mRNA expression (Figure 1C, Figure 1D). Based on the statistical differences (P value<0.0005 and P value>0.001) of patients, we divided each pair of tissues into two groups, and classified by age, gender, tumor location and tumor style (Table 1). In order to make a further demonstration, several CRC cell lines including HCT-116, HCT-15, HT-29, Caco-2, LoVo, SW480, SW620 were cultivated to examine UGT2B7 expression. NCM460, one of normal human colorectal cell lines, was used as positive control. Results indicated that the protein expression of UGT2B7 was much higher in normal colorectal cells than tumor ones (Figure 1E). In this way, we concluded UGT2B7 was repressing during CRC development.

**Table 1:** Statistics analysis of 45 pairs of adjacent normal and CRC tissues distinguished by high (P<0.0005) and low (P>0.001) expression levels of UGT2B7

Variable	Expression differences of UGT2B7		*P value for each group
	High P<0.0005N=34	Low P>0.001N=11	
**Average Age, Years**	58.40	64.09	
**Age category, Years**			
≤70	31	7	0.4999
>70	3	4	
**Gender**			
Male	21	4	0.1147
Female	13	7	
**Location**			
Rectum	23	5	0.3132
Colon	10	6	
Epityphlon	1	0	
**Style**			
Uplift	10	2	0.0519
Disk	12	5	
Ulcerative	10	3	
Others	2	1	

The value of *P was calculated via two-way ANOVA test.

Unpaired student t test and two-way AVONA test were used to calculate the P values in each group.

### Histone methyltransferase G9A and HDAC inhibitors mediate UGT2B7 up-regulation in CRC cells

**Figure 2:**
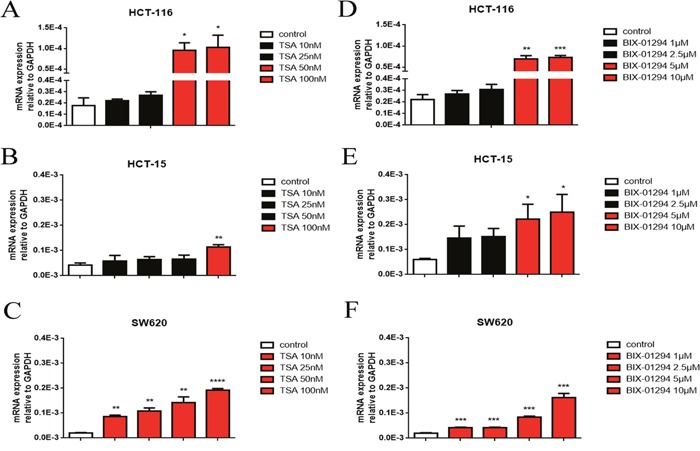
Histone methyltransferase, G9A and deacetylase, HDAC inhibitors mediate UGT2B7 induction in CRC cell lines UGT2B7 mRNA expression analyzed in CRC cell lines including **(A, D)** HCT-116, **(B, E)** HCT-15 and **(C, F)** SW620, which were treated with G9A or HDAC inhibitors, trichostatin A (TSA) and BIX-01294 for 72 hours with different dosages of 10-100 nM and 1-10μM, respectively. GAPDH was used as control to normalize the data. Results were presented from triplicated treatments and compared to the control group as means ± SEM. *P<0.05, **P<0.01, ***P<0.001, ****P<0.0001. Unpaired student t test was used to calculate the P value.

To identify whether the variants of methylated or acetylated signals in histone 3 on the promoter region of UGT2B7 may join in this phenomenon, two histone transferase inhibitors, BIX-01294 and TSA, were applied in three different CRC cell lines, HCT-116, HCT-15 as well as SW620. After treated with BIX-01294 (1-10μM) or TSA (10-100 nM) for 72h, the total RNA was extracted and then reverse transcribed. The expression of UGT2B7 was then measured by qPCR. From the data, UGT2B7 was significantly activated in each cell line (Figure 2A-2F). The induction folds for both inhibitors in HCT-116 cells were extremely higher than the others. Those manifested methylated and acetylated signals in histone 3 on the promoter of UGT2B7 were repressed in CRC.

### Morphine tolerance induces positive methylated and acetylated signals in histone 3 on the promoter of UGT2B7

**Figure 3:**
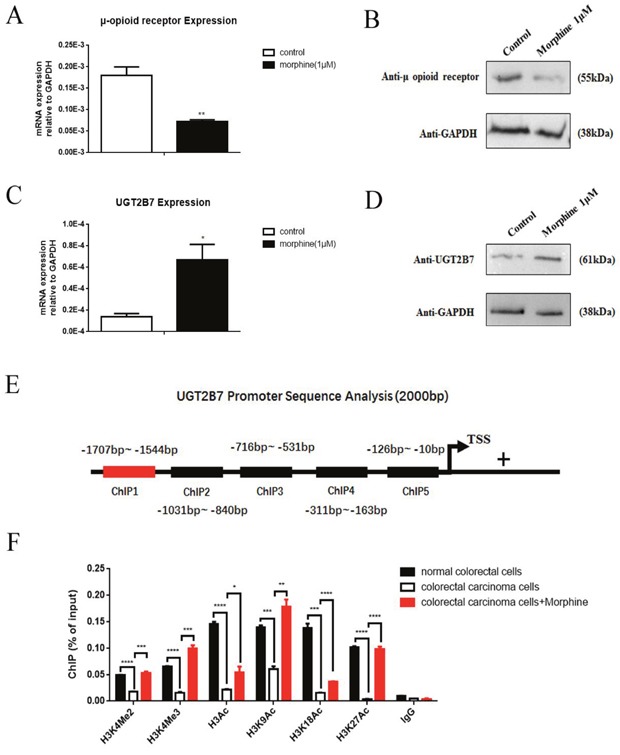
Positive methylated and acetylated signals in histone 3 regulate UGT2B7 expression in morphine tolerance-like HCT-116 cells The cells were treated with 1μM morphine for 48h to induce a tolerance status. μ-opioid receptor **(A)** mRNA or **(B)** protein expression was determined in HCT-116 cells. UGT2B7 **(C)** mRNA and **(D)** protein expression was determined in morphine tolerance-like HCT-116 cells. **(E)** Promoter Analysis of 2000 base pairs (bp) promoter sequences of UGT2B7. Five fragments covering the potential binding regions were designed and synthesized. ChIP1 primers from −1707 to −1544 were chosen and amplified. **(F)** ChIP-qPCR analysis showed the active histone modification signals in the promoter of UGT2B7. ChIP antibodies including anti-H3Ac, anti-H3K9Ac, anti-H3K18Ac, anti-H3K27Ac, anti-H3K4Me2 and anti-H3K4Me3 were performed and normalized to anti-IgG negative control group. Enrichments were calculated as percentage of total chromatin input. Results were presented from triplicated treatments as means ± SEM. *P<0.05, **P<0.01, ***P<0.001, ****P<0.0001. Unpaired student t test was used to calculate the P value.

To identify whether morphine can involve in the regulations of UGT2B7 during tolerance in CRC. HCT-116 cells were chosen and treated with 1μM morphine for 48h. The μ opioid receptor (MOR) expression was significantly declined (Figure 3A, Figure 3B). Thus, morphine tolerance-like cell models were established [20, 21]. As soon as it was generated, we checked that UGT2B7 was simultaneously activated (Figure 3C, Figure 3D). To determine whether this phenomenon was correlated to the activation of positive signals in histone 3 during morphine tolerance, we investigated each of them on UGT2B7′s promoter region via chromatin immunoprecipitation (ChIP)-qPCR assay. In brief, the primers containing five fragments distributed in 2000 base pairs (bp) of UGT2B7 upstream promoter region were designed and synthesized (Figure 3E, Supplementary Table 2). One of them with the sequence of −1707 to −1544 base pairs (bp) can be amplified so that it was applied for further tests. From the results, we noticed the positive methylated signals in histone 3 including H3K4Me2, H3K4Me3 were all receded in HCT-116 compared to normal colorectal cells, but rebounded in morphine tolerance (Figure 3F). Similar trends were also performed in integral positive signal, acetylated in histone 3 (H3Ac), including some specific ones: H3K9Ac, H3K18Ac and H3K27Ac. Among all of those, H3K4Me3 and H3K27Ac became the two signals with the biggest changes which attributed to 6- or 24- fold increases respectively in morphine tolerance-like HCT-116 cells than non-treatment control ones.

### BDNF binds with UGT2B7 promoter and attributes to its repression in CRC

**Figure 4:**
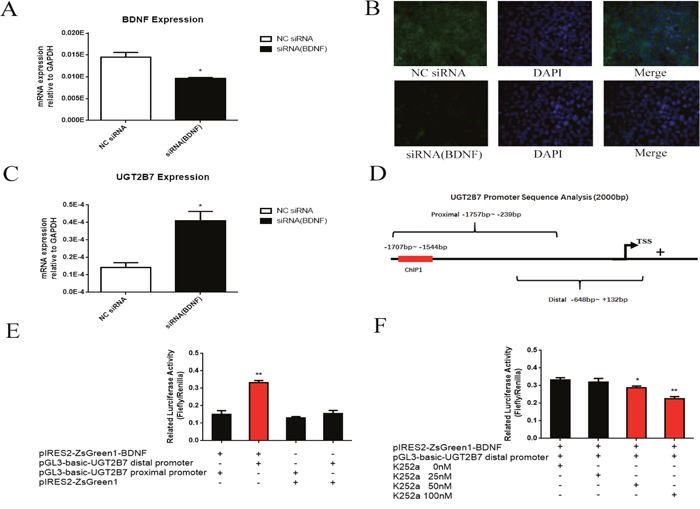
BDNF regulates UGT2B7 through promoter transactivation **(A)** mRNA expression of BDNF after knocking down BDNF in HCT-116 cells. **(B)** Immunofluorescence assay (IF) was performed to analyze the BDNF expression in HCT-116 cells after siRNA-1155 transfection. DAPI dyed cell nucleus was applied to monitor and locate the cellular targets. **(C)** mRNA expression of UGT2B7 after knocking down BDNF in HCT-116 cells. **(D)** Diagram of reporter gene sequences designed in UGT2B7 promoter region. The primers for distal promoter from −1757 to --239bp covered with the binding sequences verified by ChIP-qPCR and proximal promoter from −648 to +132bp were simultaneously amplified via PCR. **(E)** The binding between BDNF and UGT2B7 in HEK293 cells investigation via luciferase assay. Similarity like BDNF overexpressing as well as enhancer pRL-TK plasmids, the reporter gene plasmids of UGT2B7 distal and proximal promoters were meanwhile transfected into HEK293 cells for 48 hours. Blank vector plasmids were used as negative control. Luciferases ratio of firefly to ranilla (RLU) was calculate to estimate the activation ability between the two genes. **(F)** The RLU values determination after BDNF inhibitor reagent, K252-a, treatment. Results were presented from triplicated treatments as means ± SEM. *P<0.05, **P<0.01. Unpaired student t test and one-way ANOVA test were used to calculate the P value.

We further attempted to know whether BDNF which overexpresses in CRC is related to the regression of UGT2B7. Three siRNA (1155, 1314, 1768) targeted to the different regions of BDNF promoter were designed and synthesized, then primarily applied in HCT-116 cells (Supplementary Table 3, Supplementary Figure 1A, Supplementary Figure 1B). From the results, siRNA (1155) can significantly inhibit the gene and it was chosen for next assays. After knocking down BDNF in HCT-116 cells (Figure 4A, Figure 4B), UGT2B7 was inversely motivated (Figure 4C). To analyze whether BDNF could bind on the promoter of UGT2B7, distal promoter of the later one from −1757 to −239bp containing with the sequences previous selected and proximal promoter from −648 to +132bp were designed and produced by PCR and inserted into pGL3-basic vector for reporter gene plasmids establishment (Supplementary Table 2, Figure 4D). Next, we overexpressed BDNF and transfected with reporter gene plasmids into HEK293 cells, luciferase assay was performed to determine this transactivation. From the data, we found BDNF can specifically interact with the promoter of UGT2B7 (Figure 4E). Using different concentrations of K252-a which was reported to inhibit BDNF can even attenuate this interaction (Figure 4F) [22].

### Interaction between BDNF and UGT2B7 can alter the signals of H3K4Me3 and H3K27Ac on the promoter of UGT2B7

**Figure 5:**
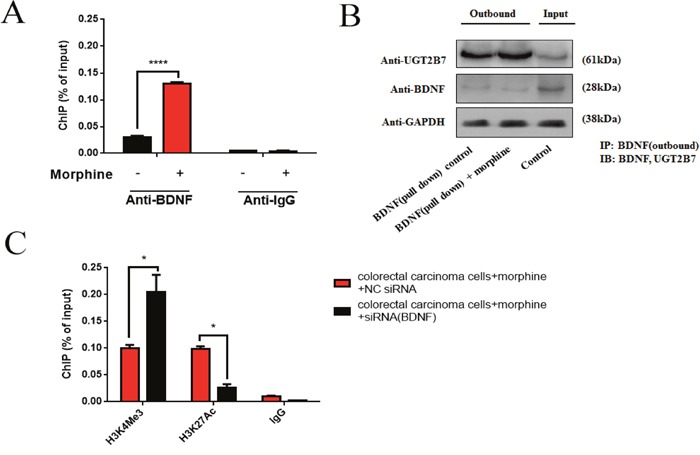
BDNF and UGT2B7 interaction induces the alterations of positive signals in histone 3 of UGT2B7 promoter **(A)** ChIP-qPCR analysis of BDNF at the UGT2B7 promoter in normal and morphine tolerance-like HCT-116 cells. Anti-IgG was performed as negative control. **(B)** Co-immunoprecipitation (Co-IP) analyzed the BDNF and UGT2B7 protein-protein interaction (PPI) in normal and morphine-tolerance HCT-116 cells. The input group was used as negative control. They were all normalized to GAPDH expression. **(C)** ChIP-qPCR analysis for the variants of active histone signals, H3K4Me3 and H3K27Ac, after knocking down BDNF in morphine tolerance-like HCT-116 cells. Anti-IgG was performed as negative control. Results were presented from triplicated treatments as means ± SEM. *P<0.05, ****P<0.0001. Unpaired student t test was used to calculate the P value.

After that, we conservatively hypothesized this interaction for BDNF and UGT2B7 may generate in the proteins. To verify that, ChIP-qPCR and co-immunoprecipitation (Co-IP) assay were carried out. Results elucidated BDNF can regulate UGT2B7 via protein-protein interaction (PPI), this binding even existed in morphine tolerance-like CRC cells (Figure 5A, Figure 5B). Interestingly, under the same condition, knocking down BDNF can further; contribute to H3K4Me3 activation and H3K27Ac suppression. Considering BDNF was opposite to UGT2B7 expression, this finding indicated H3K4Me3 should be the main signal to stimulate UGT2B7 after BDNF was knockdown (Figure 5C).

### Repression of BDNF in CRC cells gives rise to PRC1 and PRC2 selectively motivation

**Figure 6:**
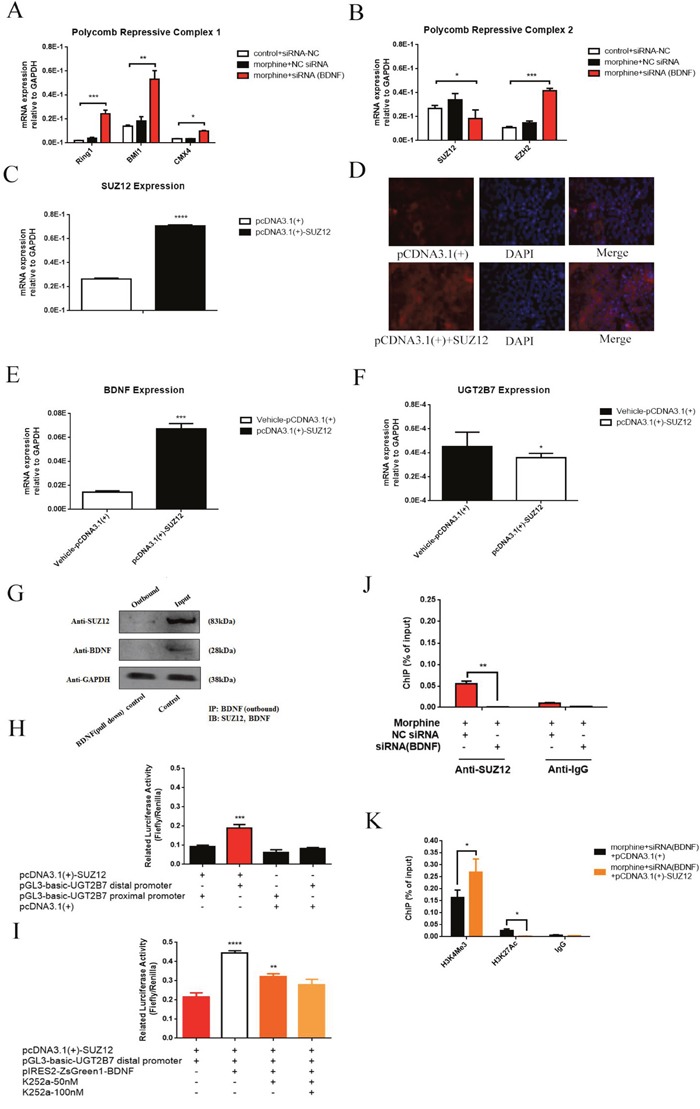
BDNF and UGT2B7 regulation is affected by core factor of PRC2, SUZ12 under morphine tolerance in CRC cells **(A)** Expression of main core factors in PRC1 including CMX4, BMl1 and Ring1B after BDNF depression in normal and morphine tolerance-like HCT-116 cells. **(B)** Expression of main core factors in PRC2 including EZH2 and SUZ12 in BDNF depression mediated normal and morphine tolerance-like HCT-116 cells. **(C)** Overexpression of SUZ12 in HCT-116 cells. **(D)** Immunofluorescence (IF) assay was used to confirm this overexpression. **(E, F)** Expression of UGT2B7 and BDNF after SUZ12 overexpression in HCT-116 cells. **(G)** Co-IP assay to determine the interaction between SUZ12 and BDNF. **(H)** Transactivation between SUZ12 and UGT2B7. Reporter gene plasmids of UGT2B7 distal and proximal promoters were transfected after overexpressing of SUZ12 in HEK293 cells and determined by luciferase assay. The plasmids of expression vehicles were added as negative control. **(I)** BDNF can further enhance SUZ12 mediated UGT2B7 transactivation. **(J)** ChIP-qPCR analysis of SUZ12 on the promoter of UGT2B7 after BDNF was knockdown in normal or morphine tolerance-like HCT-116 cells. **(K)** Variants of H3K4Me3 and H3K27Ac signals on the promoter of UGT2B7 after SUZ12 overexpression and BDNF downregulation in morphine tolerance-like HCT-116 cells. Anti-IgG was used to normalize the data as negative control. Results were presented from triplicated treatments as means ± SEM. *P<0.05, **P<0.01, ***P<0.001, ****P<0.0001. Unpaired student t test and one-way ANOVA test were used to calculate the P values.

Since H3K27Ac was alleviated on the promoter region of UGT2B7 when BDNF was knockdown in morphine tolerance CRC cells, the expression of the factors correlated to this signal such as poly-comb repressive complex (PRC) 1 or PRC2 attracted our interests for deep exploration. We examined the expression of the main core factors in PRC2, including EZH2 and SUZ12 as well as the main core factors in PRC1 containing with BMl1, CMX4 and Ring1B during BDNF recession in morphine tolerance-like HCT-116 cells. Then we found BDNF repression contributed to three core factors of PRC1 significant upregulation, but SUZ12 of PRC2 was in a converse character (Figure 6A, Figure 6B).

### SUZ12 and BDNF mutually participates in UGT2B7 regulation

To determine the role of SUZ12 in BDNF mediated UGT2B7 regulation, SUZ12 was overexpressed in HCT-116 cells (Figure 6C, Figure 6D). Preliminary, we measured the expression of UGT2B7 and BDNF then found BDNF was activated in the opposite, UGT2B7 was slightly decreased (Figure 6E, Figure 6F). Meanwhile, we noticed the reason for this phenomenon was due to the interaction of BDNF and SUZ12 (Figure 6G), the later one was able to bind with the promoter of UGT2B7 (Figure 6H). Overexpressing BDNF can enhance this transactivation (Figure 6I). Consequences of ChIP-qPCR assay illustrated that morphine tolerance had no impacts on these regulations (Figure 6J). Besides, the signal of H3K27Ac on the promoter of UGT2B7 significantly receded after SUZ12 overexpressing in BDNF depressed morphine tolerance-like HCT-116 cells (Figure 6K). All the results presented when morphine tolerance generated in CRC, PRC2 core factor, SUZ12 enrichment and BDNF disrupting would contribute to an opposite change for H3K4Me3 or H3K27Ac on the promoter of UGT2B7.

### Epigenetic inhibitors or BDNF depression influence UGT2B7 enzymatic activity and substrate regioselective catalysis alterations in morphine tolerance-like HCT-116 cells

**Figure 7:**
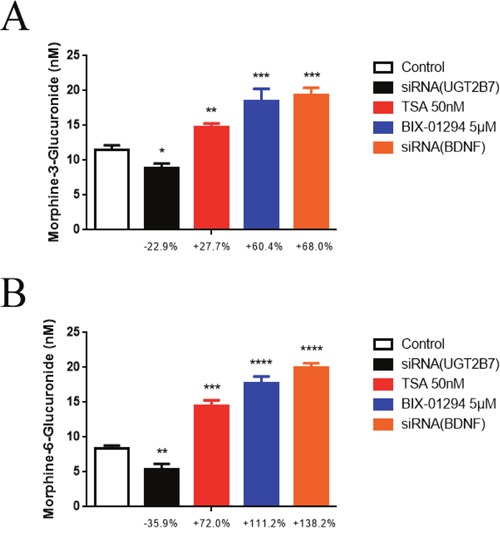
Epigenetic inhibitors and BDNF repression induce the alterations of UGT2B7 enzymatic activity and morphine regioselective glucuronidation in CRC The concentrations of **(A)** M3G and **(B)** M6G. Morphine tolerance-like HCT-116 cells which transfected with siRNAs for knocking down BDNF and UGT2B7 were continually treated with 50 nM TSA or 5μM BIX-01294 for 72 hours, respectively. All active proteins were then extracted for incubation assay *in vitro* and the samples were measured and determined by HPLC-MS/MS. NC siRNA was transfected into the cells after DMSO treatment as negative controls. Results were presented from 12-time treatments compared to the control group as means ± SEM. *P<0.05, **P<0.01, ***P<0.001, ****P<0.0001. Unpaired student t test was used to calculate the P value.

To acknowledge whether these mechanisms can change the enzymatic activity or substrate regioslective catalysis of UGT2B7, morphine glucuronidation assay was then performed *in vitro*. Firstly, siRNA-621 specific targeted to UGT2B7 was verified previously [21] and applied to the next tests (the sequences were shown in Supplementary Table 3). After that, we determined the quantities of M3G and M6G under epigenetic inhibitors BIX-01294 (5μM) and TSA (50 nM) treatment or BDNF deficient morphine tolerance-like HCT-116 cells by HPLC-MS/MS. Knocking down UGT2B7 was conducted as a negative control. Based on the data, we found the enzymatic activities of UGT2B7 were all increased after HDAC, G9A inhibitors or siRNA (BDNF) treatment (Figure 7A, Figure 7B). In contrast, the quantity ratio of M3G to M6G dropped off compared to the control group (Table 2), which means M6G selectively produced after the fortification of UGT2B7 enzymatic activity.

**Table 2:** Concentration ratios of M3G to M6G in morphine glucuronidation assay

Variable	Concentration ratio: M3G/M6G
Control	1.37
siUGT2B7	1.65
50 nM TSA	1.02
5μM BIX-01294	1.04
siBDNF	0.97

## DISCUSSION

**Figure 8:**
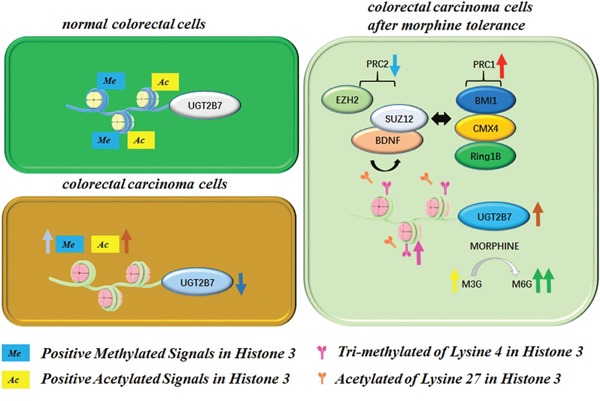
Mechanism for BDNF mediated variants of positive methylated and acetylated signals in histone 3 on the promoter of UGT2B7 during morphine tolerance in CRC

UGT2B7 is one of the main metabolism enzymes to catalyze many endogenous compounds such as retinoic acid, estriol and exogenous drugs including anti-tumor zidovudine (AZT) and epirubicin [23]. Here, we detected that it significantly repressed in the tumor tissues of CRC patients due to the cell factors mediated alterations of positive methylated or acetylated signals in histone 3, as seen in Figure 8. Two positive signals including H3K4Me3 and H3K27Ac had significantly upregulated from depression on the promoter of UGT2B7 during morphine tolerance. Accordingly, BDNF, PRC1 and PRC2 were also involved in the UGT2B7 activation. The purpose of this study was to determine whether this mechanism can finally transform the enzymatic activity of UGT2B7 then affect morphine regioselective glucuronidation, our conclusion may guide for morphine application in tumor-induced pain.

The microarray data of adjacent normal and tumor tissues from CRC patients elucidate that the depression differences of UGTB7 can be divided to high (P<0.0005) or low (P>0.001) significant difference groups. Age, gender, tumor location and tumor style are obviously correlated to these differences. According to the information, we found age would be a main element in the high difference group. The number of people in under 70-year-old group which featured with higher UGT2B7 expression differences was 10 times more than those over 70. Many clues indicate its expression is persistently raising from childhood to adolescence but significantly decreasing after 20 age-old [24, 25]. This phenomenon may give us an explanation why younger CRC patients held higher expression level of UGT2B7 in their normal colorectal tissues compared to the tumor ones.

Gradient concentrations of TSA (10-100 nM) and BIX-01294 (1-10μM) were administrated into three CRC cell lines. We found the expression of UGT2B7 was significantly activated in the cells which were treated with either of the chemical compounds. Here, these two candidate compounds used in our tests are potential drugs to remedy colorectal cancer, although they have not been approved to come into the market [26, 27]. As regular HDACs inhibitor, TSA is used for target gene revitalization by altering its histone acylation signals on its promoter and performs therapeutic effect in tumors. For instance, it can elevate the oxaliplatin renal cell carcinoma (RCC) cure sensitization during combination usage by upregulating organic cation transporter 2 (OCT2) expression [28]. Accordingly, BIX-01294 can prohibit euchromatic histone-lysine N-methyltransferase 2 (EHMT2, G9A) and G9A-like proteins then impair the transformations for methylated signals of lysis 4 or 9 on the promoter of target genes in histone 3 [29, 30]. Also, it can release the proliferation and tumorigenicity of cancer cells by leading to G9A inactivation [31]. Interestingly, H3K4Me2 regulated by human histone demethylase LSD2 plays a contrary role to H3K9Me2. So BIX-01294 can be applied for changing the former signal, H3K4Me2 which is correlated to the stable complex of G9A and LSD2 [32]. Besides, it once reported that this chemical compound reduces the signal of H3K4Me3 following by concentration- and time-dependent manner in malaria parasite [33]. For these reasons, in order to further acknowledge the functions of TSA and BIX-01294, we then checked some of the positive methylated or acetylated signals in histone 3 on the promoter of UGT2B7 in normal or CRC cell lines. Results indicated the signals of H3K4Me2, H3K4Me3 or H3K9Ac, H3K18Ac, H3K27Ac have significantly depressed in CRC compared to the normal cells. Their reductions would become one vital reason for UGT2B7 silencing in CRC.

Some substrates of UGT2B7 can even raise the enzyme gene expression such as zidovudine [34]. Similarly, morphine can upregulate its expression not only in CRC but also in the normal human liver cells [21]. Based on our results, the variants of positive acetylated or methylated signals in histone 3 on the promoter of UGT2B7 also involved in this activation. On the other hand, we were also focusing on the two prominent signals including H3K4Me3 and H3K27Ac which have 6- or 24-fold change respectively in morphine tolerance-like HCT-116 cells comparing to non-morphine treatment control group. At the same time, Knocking down BDNF decreased H3K27Ac signal but made H3K4Me3 ascending, which illustrates BDNF recession mediated UGT2B7 activation was mainly dependent on the enrichment of H3K4Me3. Since overexpressing of BDNF will repress G9A activity, besides, G9A can pass negative feedbacks to the signal of H3K4Me3, so that the above conclusion could be effectively explained [35, 36].

It is worth mentioning that H3K27Ac develops a different character on the same gene promoter region compared to H3K27Me3, the later signal which can specifically make PRC2 recruitment [37]. At the same time, PRC1 is correlated to the ubiquitination signal of histone 2A lysine 119 (H2AK119U1), which appears together with H3K27Me3 [38]. Recent research reflected core factor CBX of PRC1 participates in the recruitment mediated by the signal variants of H3K27Me3, which is namely to say that both PRC1 and PRC2 would be correlated to the signal variants of H3K27Me3 and H3K27Ac [39, 40]. In addition, the expressions of core factors were all significantly increased excepting from SUZ12 in PRC2 after knocking down BDNF during morphine tolerance in CRC. Meanwhile, SUZ12 could involve in BDNF mediated UGT2B7 silencing in CRC via further blocking the signals of H3K27Ac and increasing H3K4Me3. Since BDNF and SUZ12 can interact in the protein level based on our data, which implied both of them would be mutually enriched on the promoter of UGT2B7 and spur the gene target as the form of complex.

BDNF mediated the variants of decorating signals in histone 3 not only changes UGT2B7 expression, but also impacts its enzymatic activity and morphine regioselective glucuronidation. We discovered knocking down UGT2B7 in morphine tolerance-like HCT-116 cells, the productions of 3- or 6-site morphine glucuronides were decreased by 22.9% or 35.9% respectively compared to NC siRNA transfected control group. In contrast, they were overwhelmingly boosted during UGT2B7 activation, which shows that the role of BDNF is extremely remarkable, compared to the group of histone kinase inhibitors treatment. Our previous research indicated lithocholic acid (LCA) regulated UGT2B7 repression performs the same change to morphine glucuronidation in human liver cells as the production of M3G decreases less than M6G [21]. Besides, epirubicin can activate UGT2B7 and contribute to an increase of M6G more than M3G [41]. These reports all potently supported what we have recognized. As a consequence, along with the quantifications of two main metabolites, inducing or repressing of UGT2B7 performed regioselective catalysis to morphine.

In summary, we identified the role of BDNF in the variants of positive signals of methylated or acetylated in histone 3 on the promoter of UGT2B7 under morphine tolerance *in vitro*. The conclusions reveal that when morphine tolerance happens in CRC, inhibiting UGT2B7 expression would be beneficial to its tolerance reverse by significantly declining the productions of M6G comparing to M3G. These mechanisms may guide the morphine treatment in colorectal carcinoma-induced pain.

## MATERIALS AND METHODS

### Study design and experimental methods

All the sources of reagents, antibodies, DNA plasmids and experimental materials were listed in *Supporting Information*. We showed correlated data of normal or tumor tissues from CRC patients in Supplementary Table 1. The pairs of primers and siRNA sequences were summarized in Supplementary Table 2 and Supplementary Table 3. For the cultivation methods of each cell line as well as experience methods, for example realtime-PCR, chromatin immunoprecipitation (ChIP) and (Co-IP)co-immunoprecipitation, please referred to *Supporting Information* for detailed.

### Statistical analysis

Meta-analysis of UGT2B7 differential transcriptions in adjacent normal and tumor tissues of CRC patients were applied with unpaired student t test. Other statistics data were expressed as mean ± SEM derived from 3 or 12 paralleled independent studies and counted by the software of GraphPad Prism 6.0 (GraphPad Software Inc., San Diego, USA). Western blotting was performed in siRNA selection assay which targeted to BDNF and normalized to Intensity of optical density (IOD) values of GAPDH in each group, we used Image Pro Plus 6.0 software to determine each stripe's IOD value in the blots. We also used statistics of one-way or two-way ANOVA test as well as unpaired student t test to estimate the P values in each difference of integral experiments.

## SUPPLEMENTARY MATERIALS FIGURES AND TABLES


