# Supplementary material for: Brain-derived neurotrophic factor involved epigenetic repression of UGT2B7 in colorectal carcinoma: A mechanism to alter morphine glucuronidation in tumor
Source: Oncotarget. 2017 Mar 16;8(17):29138–50. doi: 10.18632/oncotarget.16251 (PMC5438719; doi:10.18632/oncotarget.16251)
Supplement: Supplementary file 1 [file oncotarget-08-29138-s001.pdf]

Brain-derived neurotrophic factor involved epigenetic repression of UGT2B7 in colorectal carcinoma: A mechanism to alter morphine glucuronidation in tumor

SUPPLEMENTARY DATA

MATERIALS AND METHODS

Reagents

Penicillin, streptomycin, fetal bovine serum (FBS), trypsin, McCoy's 5A, Dulbecco's modified Eagle medium (DMEM), Lipofectamine 3000 and 2-Solution DAB Kit were purchased from Life (Invitrogen Life Technologies, San Jose, CA, USA). Chromatin immunoprecipitation assay kit (P2078), Bull Serum Albumin (BSA), BCA kit, RIPA Lysis Buffer, phenylmethylsulfonyl fluoride (PMSF) were acquired from Beyotime (Beyotime Biotechnology, Nantong, China). Total RNA Extraction Kit was obtained from Axygen Company (Axygen, China). BIX-01294, Trichostatin A (TSA), Hematoxylin, K252-a were acquired from Sigma Corporation (Sigma, Sigma-Aldrich, Shanghai, China). Morphine, M3G and M6G were purchased from Cerilliant Corporation (Round Rock, TX, USA). M6G-d3, as internal standard, was labeled by Biomag System Company (Changshu, Jiangsu, China). Co-Immunoprecipitation (Co-IP) assay kit (C600689) was purchased from Sangon Company (Sangon Biotech, Shanghai, China). siRNAs and all primers for qPCR were also designed and synthesized by Sangon Company (Sangon Biotech, Shanghai, China). PCR reagents, restriction enzymes, A-tailing kit, pMD19-T vector, PrimeScript Reverse-transcription (RT) Kit (Perfect Real Time) and SYBR Premix Ex Taq (Tli RNaseH Plus) were purchased from Takara Company (Takara, Japan). Luciferase assay kit was bought from Promega Company (Madison, WI, USA). ECL Western Blotting Substrate (Biomiga, San Diego, USA).

Antibodies

Rabbit polyclonal antibody against μ-opioid receptor (MOR) (ab10275), UGT2B7 (ab126269), H3K4Me2 (ab7766), H3K4Me3 (ab8580), H3Ac (ab47915), H3K9Ac (ab10812), H3K18Ac (ab1191), H3K27Ac (ab4729) and Alexa Fluor 488 goat anti-rabbit IgG (H+L) antibody (ab150077) were all purchased from the Abcam Company (Shanghai, China). Normal rabbit polyclonal antibody against IgG (sc2027) was purchased from Santa Cruz (Santa Cruz Biotechnology, USA). Rabbit polyclonal antibody against SUZ12 (CatalogNo:20366-1-AP), BDNF (CatalogNo:25699-1-AP) were bought from Proteintech Company (Proteintech Group, Inc, IL, USA). Mouse monoclonal antibody against glyceraldehyde phosphate dehydrogenase (GAPDH) was purchased from KangChen Bio-tech Inc (Shanghai, China). HRP-conjugated goat anti-rabbit IgG(H+L) or HRP-conjugated goat anti-mouse IgG(H+L) was obtained from MultiSciences Biotech Co, Ltd (Hangzhou, China).

DNA plasmids

pCDNA3.1(+), pIRES2-ZsGreen1 as control vehicle, pCDNA3.1(+)-SUZ12, pIRES2-ZsGreen1-BDNF as overexpressing plasmids were sequenced and bought from YouBio Corporation (YouBio Tech, Changsha, China). pRL-TK as luciferase enhancer plasmid, pGL3-basic as control vehicle plasmid which were used for luciferase assay was obtained from Promega Company (Madison, WI, USA). pGL3-basic-UGT2B7 distal promoter containing with the targeted sequences in ChIP assay and pGL3-basic-UGT2B7 proximal promoter (Supplementary Table 2) plasmids were designed and established by PCR, after A-T cloning to pMD19-T vector and double digesting, it was ligated to pGL3-basic vector then sequenced by Sangon Company (Sangon Biotech, Shanghai, China).

Experimental materials

HPLC-grade methanol and formic acid were purchased from Tedia Company (Fairfield, OH, USA). Ultrapure water (18.2 MΩ) was obtained from an ELGA–pure lab Ultra system (High Wycombe, UK). Agilent HILC PLUS SB-C18 column (2.1 mm × 50 mm, 3.5μm, Agilent, USA). Data acquisition and processing were performed using Analyst 1.5.2 (AB SCIEX). All centrifugations were performed on an Eppendorf 5415R Refrigerated Micro-centrifuge (Eppendorf, Germany).

Cell cultivation and compounds incubation

All the human cell lines were cultured at an atmosphere of 5% CO_2 and 95% air at 37°C and passaged by 0.25% EDTA mixed trypsin. HCT-116, HCT-15, HT-29 cell lines were cultivated by McCoy's 5A; NCM460 (human normal colorectal cell line), LoVo, SW480, SW620, Caco-2 and HEK293 were all cultivated by Dulbecco's Modified Eagle's Medium (DMEM). Each medium was added 10% fetal bovine serum (FBS), 1% penicillin, streptomycin. After the cells growing to 70% convergence degree, the drugs or chemical compounds

can be added. In brief, before they were diluted with DMSO and added into DMEM medium, all the chemical compounds were incubated for a period of indicated time. Next, we changed the old medium to fresh one containing different concentrations of compounds every 24h. Same volumes of DMSO (less than 0.1%) were treated the cells as control.

DNA plasmids and siRNA transfection

HCT-116 cell line was primary seeded in 6-well plates at a density of 0.5×10^6 per well and pre-cultured to 60% confluence. Then, 2μg DNA plasmid mixed with P3000 reagent or 400nM siRNA were treated in non-serum McCoy's 5A medium combining with the reagent of Lipofectamine 3000 and incubated 5min at room temperature. After that, all the mixture solutions were subjected to the cells. Experiencing 5.5 hours' incubation, the serum-free medium was changed by complete medium including different chemical compounds and culturing for another 48h (transfer the medium again at 24h). Then all the cells can be harvested for further experiment.

Luciferase assay

HEK293 cell line was first seeded in 24-well plate at a density of 0.5×10^5/well and pre-cultured to 60% confluence. In each hole of plate, we transfected 500ng expression plasmid, 250ng report gene plasmid and 50ng pRL-TK SV40 enhancer plasmid based on the methods we referred to previously [28]. Then we added different chemical compounds in complete medium after 5.5 hours` incubation, and repeated this operation every 24h. After 48 hours` cultivation, the cells were harvested and subjected to the luciferase assay using chemiluminescence apparatus (Promega GloMax 96) combing with dual-luciferase reporter mode in its system according to the manuscript protocol. Relative light unit (RLU) value in each sample was normalized to the ratio of firefly luciferase activity from the promoter of UGT2B7 to renilla luciferase.

Chromatin immunoprecipitation (ChIP) assay

ChIP assay was carried out as the procedures provided by the manufacturer of kit. The breakage of UGT2B7 genome by Bioruptor UCD200 ultrasonic cell disruptor (Belgium) was primary decided as sizes 400-800bp through agarose gel electrophoresis (AGE). Then all the samples were all repeated this ultrasound method again for next immunoprecipitation and purification assays. Realtime-PCR was applied to do a further analysis. The enrichment of each histone modification signal was indicated as percentage of input. Pulling down the protein IgG was used as negative control to normalize other signals` alteration.

Ethics

All the human adjacent normal and CRC tumor tissues were collected from the Specimen Bank of Zhejiang Cancer Hospital (Hangzhou, China) and strictly conducted in accordance with the protocols approved by the Institutional Review Board of Zhejiang Cancer Hospital. The paperwork was according to the documentation of 'The Detailed Rules and Regulations of Medical Animal Experiments Administration and Implementation' (Document No. 1998–55, Ministry of Public Health, China).

Reverse transcription-PCR (RT-PCR) and quantitative realtime-PCR

Total RNA was extracted from human tissues or cells and purified by kit. Then RNA was reverse transcribed to cDNA via PrimeScript RT Master Mix (Takara). After each sample was mixed with SYBR Premix EX Taq (Takara), the quantitative realtime PCR was performed to determine each target gene expression via specific primers (Supplementary Table 3). The relative expression of transcripts was quantified and normalized to the expression of housekeeping gene of PPIA for tissues and GAPDH for cells.

Co-immunoprecipitation (Co-IP) and western blotting assay

The Co-IP assay was performed following the manual of the kit. In brief, we extracted the total proteins from the cell lysates containing RIPA and 1×PMSF reagents, then added 0.7mL cell lysate solution with specific antibodies and Protein A/G Plus Agarose (kit, Beyotime) to rotate overnight. After eluting by Co-IP buffer several times and mixing with protein loading buffer supplied in the kit, all the samples were subjected to SDS-PAGE. For western blotting assay, 10-50μg protein samples measured by BCA assay in different tests were subjected for running the electrophoresis. PVDF membranes were used to transfer the proteins from gels for 2h, 200mA. Soon after that, the blots were merged in blocking solution containing 5% non-fat milk in TBST buffer (100mM Tris-HCl, pH7.4, 150mM NaCl and 0.1% Tween 20) for 1h at room temperature. Following, primary antibodies including UGT2B7(1:1000), BDNF (1:2000), SUZ12 (1:2000), GAPDH (1:2000), μ-opioid receptor (MOR, 1:500) were mixed with TBST and incubated to the blots overnight at 4°C. After washing three times to remove the primary antibody, the HRP-conjugated goat anti-rabbit or anti-mouse IgG (H+L) (1:2000) was used as secondary antibody for detection by ECL system. Target proteins were visualized to expose the membranes through G-box chemiluminescence imaging (Syngene). GAPDH

was paralleled as an internal reference to control the expression of different target proteins.

Immunohistochemistry (IHC) assay

Normal or tumor tissues of CRC patients were fixed in 4% paraformaldehyde for 12h then preserved in 30% cold sucrose mixed with PBS solution overnight. Each section of tissue (100μm) were cut by freezing microtome and immerged in 0.3% H_2O_2-PBS solution for eliminating the endogenous peroxidase. Soon after that, they were incubated in 10% goat serum (Santa Cruz, Los Angeles, CA, USA) in PBS, and primary UGT2B7 rabbit polyclonal antibodies (diluted with 1:1000) overnight at 4°C before HRP-conjugated secondary goat anti-rabbit polyclonal antibody (1:2000) incubated for 1h. Following, they were treated with a peroxidase substrate solution, 3, 3'-diaminobenzidine tetra hydrochloride (DAB; Vector Laboratories) before counterstaining reagent, hematoxylin (Sigma, Sigma-Aldrich, Shanghai, China) were applied. The prepared slides were observed with a 400× of magnification under an Olympus BX41 microscope to measure the expression of UGT2B7. Negative control group without primary antibody was carried out with the same procedure as described above in the preliminary experiments.

Cell immunofluorescence assay

Cells were cultured on the glass slides of 6-wells and transfected with siRNAs or DNA plasmids as previously referred. Then, they were fixed by stationary liquid at 4°C overnight. Before thrice cleanings by PBST (1%Triton X-100 mixed with PBS, pH7.4), the glass slides were immerged into 5% non-fat milk in TBST buffer (100mM Tris-HCl, pH7.4, 150mM NaCl and 0.1% Tween 20) for 1h at room temperature. Following by incubating with BDNF or SUZ12 primary rabbit polyclonal antibody (1:100) TBST buffer for 4°C overnight and thrice PBST washes, Alexa Fluor 488 goat anti-rabbit IgG (H+L) antibody was then used to incubate the slides for 1h at room temperature. Finally, each target gene expression was examined under fluorescence microscope at 400× magnification. Nuclei were visualized and located by staining the dye of 4',6-Diamidino-2-Phenylindole, Dihydrochloride (DAPI, Life Company).

Glucuronidation assay

Cells were primarily treated with 1μM morphine for 48h to induce cell tolerance status then transfected with siRNAs or treated with different chemical compounds at 80% confluence. We lysed the cells and extracted the total proteins. According to the protocols published previously [41], all the proteins were quantified by BCA assay and extracted 0.1mg/ml to incubate with 500μM morphine. 2μl $HClO_4$ (70%, v/v) was used to terminate the reaction. After that, acetonitrile was added to precipitate the protein in the samples at a ratio of 2:1 of solvent. After centrifuging at 13,000g for 10 min, the supernatant was separated. M6G-d3 (internal standard) was added into the supernatant with a final concentration of 10ng/ml in each sample. Next, the collected liquid was evaporated and dried in the centrifugal thickener (LABCONCO Company) for HPLC-MS/MS determination.

HPLC-MS/MS determination

The HPLC-MS/MS method for determining the concentration ratios of M3G and M6G was established previously [21]. Based on that, we acquired the data of morphine glucuronidation and made further estimation to the enzymatic activity and substrate regioselectivity of UGT2B7.

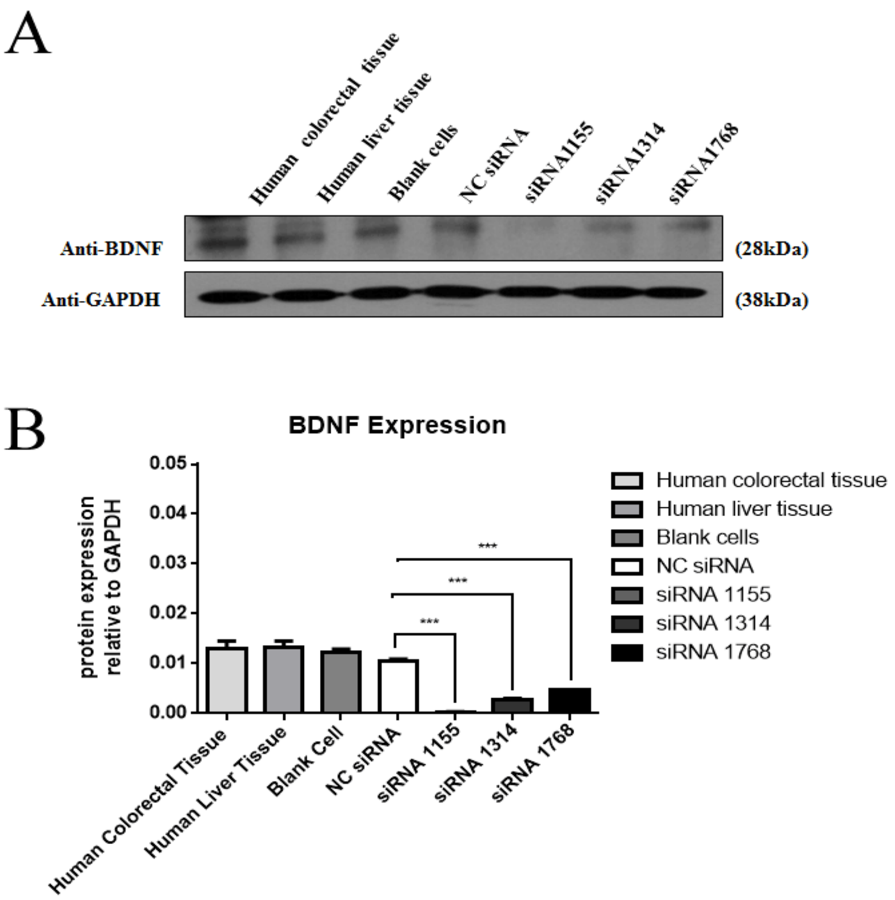

Supplementary Figure 1: siRNAs functional verification and selection for BDNF. (A) Western blotting assay was performed to determine the protein expressions of UGT2B7 and BDNF after siRNA transfected into HCT-116 cells. GAPDH was used as control to normalize the data. For BDNF, three siRNAs targeting to it were designed and synthesized, including siRNA-1155, 1314 and 1768. **(B)** Intensity of optical density (IOD) values were measured and normalized to GAPDH for each strip in the blots. Both human colorectal and liver tissues were used as positive control; Proteins of blank cells were used as blank control. NC siRNA was transfected as negative control. Results were presented from triplicated treatments as means ± SEM. ***$P<0.001$. Unpaired student t test was used to calculate the P value.

Supplementary Table 1: The information of tissue specimens in CRC patients

NUMBER	GENDER	AGE	LOCATION	TNM stage
1	Male	36	RECTUM	T4N0M0
2	Male	73	EPITYPHLON	T3N0M0
3	Male	64	RECTUM	T4N2M0
4	Female	56	RECTUM	T4N1M0
5	Male	52	RECTUM	T4N1M0
6	Male	53	RECTUM	T3N0M0
7	Male	78	RECTUM	T3N0M0
8	Male	60	RECTUM	TisNOMO
9	Male	52	RECTUM	T4N2M0
10	Male	57	COLON	T3N2M1
11	Female	69	RECTUM	T2N0M0
12	Female	66	RECTUM	T2N0M0
13	Male	47	COLON	T4N1M0
14	Male	66	RECTUM	T4N0M0
15	Female	52	COLON	T3N0M0
16	Male	60	RECTUM	T4N0M0
17	Male	64	COLON	T4N1M0
18	Male	69	COLON	T3N1M0
19	Female	61	RECTUM	T4N1M0
20	Male	64	CECUM	T4N1M0
21	Female	79	RECTUM	T4N1M0
22	Female	62	RECTUM	T2N0M0
23	Female	61	RECTUM	T4N1M0
24	Male	44	COLON	T3N0M0
25	Female	68	RECTUM	T4N2M1
26	Male	60	RECTUM	T4N0M0
27	Female	51	RECTUM	T4N2M0
28	Female	45	COLON	T4N1M0
29	Female	44	RECTUM	T2N0M0
30	Female	72	RECTUM	T2N0M0
31	Female	52	CECUM	T4N0M0
32	Male	45	RECTUM	T4N2MO
33	Female	53	RECTUM	T3N1M1
34	Female	80	COLON	T4N0M0
35	Male	45	COLON	T4N0M0
36	Male	81	RECTUM	T2N1MO
37	Male	52	COLON	T4N0M0
38	Female	73	COLON	T4N0M0
39	Female	57	COLON	T4N0M1
40	Male	63	COLON	T4N0M1
41	Female	63	RECTUM	T4N2M1
42	Male	60	RECTUM	T4N1M0
43	Male	58	RECTUM	T2N1M0
44	Female	53	RECTUM	T4N2M1
45	Male	69	COLON	T3N0M0

Supplementary Table 2: The sequence information for each pair of primers used in our tests

VARIABLE	SEQUENCES
Realtime PCR	
UGT2B7	AAGAAAGGGCCAACGTAATT (FORWARD)
108bp NM_001074	AGAGCCGAGTATTGAGACCTAA (REVERSE)
BDNF	CTTTCTGCTGGAGGAATACAA (FORWARD)
131bp NM_170735	CCGCCGTTACCCACTCACTA (REVERSE)
SUZ12	CAGCTCATTTGCAGCTTACGT (FORWARD)
168bp NM_015355	TACCTGTGGGAACTTGCCTTA (REVERSE)
EZH2	CAGAGTACATGCGACTGAGACA (FORWARD)
114bp NM_004456	CATGGTTTAAGATTTCCGTTC (REVERSE)
CMX4	GCTGATCGCCTTCCAGAAC (FORWARD)
89bp NM_003655	CCTGCACCACTAGCGGTTT (REVERSE)
BMI1	GATGCTGCCAATGGCTCTAAT (FORWARD)
127bp NM_005180	CTTTCCGATCCAATCTGTTCT (REVERSE)
Ring1B	TGAGGAGCCTGCTTTGCC (FORWARD)
68bp NM_002931	AGGTGCGATGTAGATGGTGTA (REVERSE)
GAPDH	CATGAGAAGTATGACAACAGCCT (FORWARD)
113bp NM_002046	AGTCCTTCCACGATACCAAAGT (REVERSE)
PPIB	AAGTCACCGTCAAGGTGTATTTT (FORWARD)
153bp NM_000942	TGCTGTTTTTGTAGCCAAATCCT (REVERSE)
RT-PCR	
UGT2B7	GCCACATACATTAACCCACCCTTTCA (FORWARD)
DISTAL promoter	TATATTTTAGAAGAGTGTCCAGAAC (REVERSE)
1518bp	
UGT2B7	
PROXIMAL	AAAGAAGCCAGTCACAAAATACCAC (FORWARD)
promoter	CCACACCAGCACCTTTCCACAAT (REVERSE)
780bp	
ChIP-qPCR	
UGT2B7-ChIP1	GCTGTCAGATAGCATTTTACCCATA (FORWARD)
164bp	GAGATGGAATCTACTCCTGGTGAAG (REVERSE)
UGT2B7-ChIP2	CAGCCGAGTCAGTAGTTTTAGCAG (FORWARD)
192bp	GTTAGCTTGTTCTCCGACTCACTTC (REVERSE)
UGT2B7-ChIP3	TGATGGTTGTAAGAATCAACATGTA (FORWARD)
180bp	CCTGTCCTAGGTAATTGCTAATTG (REVERSE)
UGT2B7-ChIP4	TAGTTTTGTGTCAAATGGACTGC (FORWARD)
149bp	CCCTTTTATGTTTATGAGTGCTATC (REVERSE)
UGT2B7-ChIP5	TCATTTGTCTCTTTGCCATCC (FORWARD)
117bp	TGGTTATGTCCAAAGATAAATTAGC (REVERSE)

Supplementary Table 3: The sequence information of each pair of siRNAs used in our tests

VARIABLE	SEQUENCES
BDNF	
siRNA (1155)	Sense: GCAAACAUCCGAGGACAAGTT
	Antisense: CUUGUCCUCGGAUGUUUGCTT
siRNA (1314)	Sense: GUUCGGCCCAAUGAAGAAATT
	Antisense: UUUCUUCAUUGGGCCGAACTT
siRNA (1768)	Sense: GGCGAUUCAUAAGGAUAGATT
	Antisense: UCUAUCCUUAUGAAUCGCCTT
UGT2B7	
siRNA (621)	Sense: CCUACGUACCUGUUGUUAUTT
	Antisense: AUAACAACAGGUACGUAGGTT
Negative control (NC) siRNA	
siRNA	Sense: UUCUCCGAACGUGUCACGUTT
	Antisense: ACGUGACACGUUCGGAGAATT
